# Impact of Chronic RAAS Use in Elderly COVID-19 Patients: A Retrospective Analysis

**DOI:** 10.3390/jcm10143147

**Published:** 2021-07-16

**Authors:** João Oliveira, Joana Gameiro, João Bernardo, Filipe Marques, Cláudia Costa, Carolina Branco, Inês Duarte, José Fonseca, Carolina Carreiro, Sandra Braz, José António Lopes

**Affiliations:** 1 Department of Nephrology and Renal Transplantation, Centro Hospitalar Universitário Lisboa Norte, 1649-028 Lisboa, Portugal; joana.estrelagameiro@gmail.com (J.G.); joaofbernardo@gmail.com (J.B.); filipedcmarques@campus.ul.pt (F.M.); claucosta_30@hotmail.com (C.C.); carolinagbranco@hotmail.com (C.B.); ines.cc.duarte@gmail.com (I.D.); jose.nuno.agapito@gmail.com (J.F.); jalopes93@hotmail.com (J.A.L.); 2 Department of Internal Medicine, Centro Hospitalar Universitário Lisboa Norte, 1649-028 Lisboa, Portugal; ctc.1991@gmail.com (C.C.); sandrabbraz@gmail.com (S.B.)

**Keywords:** elderly, COVID-19, renin–angiotensin–aldosterone system, mortality

## Abstract

Corona Virus Disease-19 (COVID-19) recently emerged as a global pandemic. Advanced age is the most important risk factor for increased virus susceptibility and worse outcomes. Many older adults are currently treated with renin–angiotensin–aldosterone system (RAAS) inhibitors and there is concern that these medications might increase the risk of mortality by COVID-19. This is a retrospective cohort of 346 patients older than 65 years with COVID-19, at the Department of Medicine of the Centro Hospitalar Universitário Lisboa Norte, in Portugal, hospitalized between March 2020 and August 2020. Mean age was 80.9 ± 8.7 years old. Most patients had arterial hypertension (n = 279, 80.6%), almost half (n = 161, 46.5%) had cardiovascular disease and approximately one-third of patients had heart failure (n = 127, 36.7%) or diabetes Mellitus (n = 113, 32.7%). Ninety-eight patients (28.3%) had chronic kidney disease and almost half of the patients (49.4%) were chronically under renin–angiotensin–aldosterone system (RAAS) inhibitors. Twenty percent of patients died during hospitalization. In a multivariate analysis, older age (OR 1.11, 95% CI 1.04, 1.18, *p* = 0.002), absence of baseline medication with RAAS inhibitors (OR 0.27, 95% CI 0.10, 0.75, *p* = 0.011), higher serum ferritin (OR 1.00, 95% CI 1.00, 1.00, *p* = 0.003) and higher lactate levels (OR 1.08, 95% CI 1.02, 1.14, *p* = 0.006) were independent predictors of mortality. Older age, higher serum ferritin and lactate levels at admission were found to be independent predictors of mortality and might act as early predictors of worsening disease in clinical practice. Chronic treatment with RAAS inhibitors appeared to be protective, supporting guidelines in not discontinuing such drugs.

## 1. Introduction

Corona Virus Disease-19 (COVID-19), caused by the Severe Acute Respiratory Syndrome Corona Virus 2 (SARS-Cov-2), recently emerged as a global pandemic. Since the outbreak, it infected 177,866,160 people and killed 3,857,974, as of 10 June 2021 [1].

Several conditions are associated with increased virus susceptibility and worse outcomes, advanced age being the most important. In elderly patients, mortality associated with COVID-19 varies widely among different countries, but patients older than 65 consistently comprise the majority of total deaths [2,3,4,5]. Mortality, in this subset of the population, is reported to be 8–36%, depending on the country and age interval analyzed [2,3,4,5,6]. Older age and coexisting comorbidities, particularly cardiovascular disease (CVD), are among the most implicated risk factors for adverse outcomes of COVID-19 [2,7,8,9,10]. As comorbidities often increase with age, older adults with multiple comorbidities are at higher risk for adverse outcomes [11].

A great proportion of adults with hypertension, diabetes Mellitus and/or chronic kidney disease (CKD) are currently treated with angiotensin-converting enzyme (ACE) inhibitors or angiotensin II receptor blockers. Concern was raised that the use of these medications would further increase risk of infection, with a more severe clinical course and risk of mortality in such patients [12].

In this study, we aim to analyze elderly patients hospitalized with COVID-19 and evaluate the relationship between renin–angiotensin–aldosterone system (RAAS) inhibitors and mortality.

## 2. Materials and Methods

This is a retrospective cohort of hospitalized patients admitted to a Dedicated Unit for COVID-19 patients (UICIVE) at the Department of Medicine of the Centro Hospitalar Universitário Lisboa Norte (CHULN), in Lisbon, Portugal, between March 2020 and August 2020. This study was approved by the Ethical Committee, in agreement with institutional guidelines. Informed consent was waived, given the retrospective and non-interventional nature of the study.

We selected as eligible all elderly patients (≥65 years of age) who tested positive by polymerase chain reaction testing of a nasopharyngeal sample for COVID-19 and were admitted at the UICIVE from March 1st to August 31st 2020. Only the first hospitalization was analyzed in patients with multiple admissions.

Data were collected from individual electronic clinical records. The following variables were analyzed: patient demographic characteristics (age, gender); comorbidities (diabetes mellitus, hypertension, chronic obstructive pulmonary disease (COPD), CVD, cirrhosis, heart failure, CKD and/or active malignancy); current treatment with RAAS inhibitors (angiotensin converting enzyme inhibitor or angiotensin receptor blockers); disease severity, according to the Brescia-COVID Respiratory Severity Scale (BCRSS) at admission; [13] laboratory values at admission (serum hemoglobin, neutrophil to lymphocyte ratio, serum albumin, serum ferritin, serum creatinine (sCr), C-reactive protein (CRP), arterial blood gas and pH analysis, lactate, lactic acid dehydrogenase (LDH)); exposure to nephrotoxins during the first week of admission (non-steroidal anti-inflammatory drugs (NSAIDS), radiocontrast, vancomycin, aminoglycosides); need for intensive care unit (ICU) admission; need for mechanical ventilation; vasopressor use; treatment options used for COVID-19 (hydroxychloroquine, lopinavir/ritonavir, corticosteroids, tocilizumab and remdesivir).

Diagnosis of COVID-19 was based on a positive polymerase chain reaction testing of a nasopharyngeal sample for COVID-19.

Presence of CKD was estimated according to the baseline sCr as an estimated glomerular filtration rate (GFR) of lower than 60 mL/min/1.73 m^2^. Acute kidney injury was diagnosed according to Kidney Disease Improving Global Outcomes (KDIGO) criteria [14]. Diabetes mellitus was diagnosed according to the American Diabetes Association criteria. [15] Hypertension was diagnosed according to the 2018 European Society of Cardiology (ESC) and European Society of Hypertension Guidelines [16]. COPD comprised emphysema and chronic bronchitis. CVD was considered whenever a history of cerebrovascular disease, chronic heart failure of any cause, cardiac ischemic disease and/or peripheral arterial disease was documented. Acidemia was defined as blood gas PH < 7.35 [17] N/L ratio at admission was calculated as: neutrophil count/lymphocyte count.

The analyzed outcome was in-hospital mortality.

Categorical variables were described as the total number and percentage for each category, whereas continuous variables were described as the mean ± standard deviation. Continuous variables were compared with the Student’s *t*-test and categorical variables were compared with the Chi-square test. All variables underwent univariate analysis to determine statistically significant factors which may have contributed to in-hospital mortality. Subsequently, variables with a significant statistical difference were included in the multivariate analysis using the logistic regression method. Data were expressed as odds ratios (OR) with 95% confidence intervals (CI). Statistical significance was defined as a *p* value <0.05. Statistical analysis was performed with the statistical software package SPSS for Windows (version 21.0).

## 3. Results

During the study period, a total of 346 elderly patients diagnosed with COVID-19 were admitted to the Department of Medicine of Centro Hospitalar Universitário Lisboa Norte.

Patients’ characteristics are presented in Table 1. Mean age was 80.9 ± 8.7 years old and almost half of the patients were male (n = 164, 47.4%). Most patients had arterial hypertension (n = 279, 80.6%), almost half (n = 161, 46.5%) had CVD and approximately one-third of the patients had heart failure (n = 127, 36.7%) or diabetes Mellitus (n = 113, 32.7%). Ninety-eight patients (28.3%) had CKD, of which 15 (15.3%) were on chronic dialysis. Mean baseline sCr was 1.0 ± 0.5 mg/dL, eGFR was 65.3 ± 21.4 mL/min/1.73 m^2^. Almost half of the patients (49.4%) were chronically under RAAS inhibitors. 

Eleven patients (3.2%) presented with a Brescia score greater than two.

Approximately half of the patients presented with anemia (49.7%) and hypoalbuminemia (54.9%). Mean sCr at admission was 1.5 ± 1.4 mg/dL, hemoglobin was 12.3 ± 2.3 g/dL, N/L ratio 7.0 ± 6.5, serum ferritin was 1113.1 ± 1731.4 µg/dL, CRP was 10.4 ± 10.0 mg/dL, lactates were 15.0 ± 10.4 mg/dL and LDH was 333.3 ± 171.5 mg/dL. Intensive care unit (ICU) admission was required in 19.7% of patients and 12.4% needed renal replacement therapy (RRT) for acute kidney injury. Vasopressors were required in 3.8% of patients and mechanical ventilation in 11.6% of patients.

The antiviral combination of lopinavir/ritonavir was the most frequent used agent (33.5%), followed by hydroxychloroquine (26.3%) and corticosteroids (24%). Remdesivir was used in 35 patients (7.2%) and tocilizumab only in four (1.2%).

Mean length of hospital stay was 34.3 ± 46.3 days and was not associated with mortality.

### 3.1. Use of RAAS Inhibitors

Almost half of patients (n = 171, 49.4%) were on either angiotensin converting enzyme inhibitor or angiotensin receptor antagonist. Characteristics are listed in Table 2.

Patients on such medications were more likely to have hypertension (91.8% versus 67.4%, *p* = 0.000), diabetes (38.6% versus 26.9%, *p* = 0.029), COPD (18.7% versus 9.7%, *p* = 0.021) and were less likely to be on dialysis (1.2% versus 7.4%, *p* = 0.009), although there were no differences in the presence of CKD. No differences were found in the presence of other comorbidities, namely, CVD, heart failure, cirrhosis and neoplasia. Age and gender were similar between populations.

Mortality was greater among patients not on RAAS inhibitors (24% versus 15.8%, *p* = 0.043). No differences were noted regarding age, gender, laboratory parameters at admission or other characteristics.

### 3.2. Mortality

Twenty percent of patients died during hospitalization.

Patients who died were older (84.8 ± 8.8 years versus 79.8 ± 8.4 years, *p* = 0.000; unadjusted OR 1.08, 95% CI 1.04, 1.11, *p* = 0.000) and were more likely to have CVD (58.3% versus 43.4%, *p* = 0.024; unadjusted OR 1.82, 95% CI 1.08, 3.09, *p* = 0.025), heart failure (48.6% versus 33.6%, *p* = 0.019; unadjusted OR 1.87, 95% CI 1.11, 3.17, *p* = 0.019), malignancy (23.6% versus 13.1%, *p* = 0.028; unadjusted OR 2.04, 95% CI 1.07, 3.90, *p* = 0.030) and CKD (44.4% versus 24.1%, *p* = 0.000; unadjusted OR 2.73, 95% CI 1.58, 4.72, *p* = 0.000), although there were no differences in CKD under dialysis. In addition, these patients were less likely to be chronically medicated with RAAS inhibitors (37.5% versus 52.6%, *p* = 0.004; unadjusted OR 0.58, 95% CI 0.34, 0.99, *p* = 0.045).

At admission, they had lower Hb levels (11.68 ± 2.54 g/dL versus 12.46 ± 2.14 g/dL, *p* = 0.009; unadjusted OR 0.86, 95% CI 0.76, 0.96, *p* = 0.010), greater N/L ratio (9.13 ± 7.64 versus 6.38 ± 6.10, *p* = 0.001; unadjusted OR 1.06, 95% CI 1.02, 1.09, *p* = 0.003) and serum ferritin (2068.37 ± 3331.75 µg/dL versus 850.03 ± 713.14 µg/dL, *p* = 0.000; unadjusted OR 1.00, 95% CI 1.00, 1.00, *p* = 0.003), greater LDH (385.77 ± 267.17 mg/dL versus 319.82 ± 134.02 mg/dL, *p* = 0.004; unadjusted OR 1.00, 95% CI 1.00, 1.00, *p* = 0.014) and lactate levels (19.22 ± 16.97 mg/dL versus 13.87 ± 7.36 mg/dL, *p* = 0.000; unadjusted OR 1.05, 95% CI 1.02, 1.08, *p* = 0.001). 

As presented in Table 3, in a multivariate analysis, older age (OR 1.11, 95% CI 1.04, 1.18, *p* = 0.002), absence of baseline medication with RAAS inhibitors (OR 0.27, 95% CI 0.10, 0.75, *p* = 0.011), higher serum ferritin (OR 1.00, 95% CI 1.00, 1.00, *p* = 0.003) and higher lactate levels (OR 1.08, 95% CI 1.02, 1.14, *p* = 0.006) were independent predictors of mortality.

## 4. Discussion

In this study of elderly patients hospitalized with COVID-19, older age, higher ferritin and higher lactate levels at admission were predictors of mortality. In contrast, we demonstrated that previous therapy with RAAS inhibitors appeared to be protective against mortality in this setting (15.8% versus 24%, *p* = 0.043; adjusted OR 0.27 (0.10–0.75), *p* = 0.011).

Advanced age has been postulated to be the single most important risk factor for mortality associated with COVID-19 [10,18,19,20]. Patients older than 80 show a greater risk of death than younger patients [20]. Data from China and Italy report that more than 50% of fatalities occur in patients older than 50 years of age [21].

An age-related decline in the small airway clearance of inhaled particles, caused by a gradual decrease in the number of cilia, may explain the high prevalence of respiratory symptoms in the elderly and their increased susceptibility to acquire infection [22,23,24]. It is believed that immunosenescence might also play a role in the increased mortality that is seen among elderly patients with COVID-19 [25,26,27]. The production of T and B cells decreases, innate immune cell function is impaired and there is an uncoordinated progression to an adaptive immune response in advanced age [27]. In addition to decreased viral clearance, this might also trigger a dysregulated immune response, resulting in a cytokine storm [28], a known characteristic responsible for worsening the clinical scenario of COVID-19 patients. Furthermore, inflammaging, a state of chronic subclinical production of inflammatory mediators and cytokines typical of the elderly, is thought to account for the poorer outcomes encountered in this population [24,29,30].

In a large series from northern Italy, case fatality rate in patients with at least 65-years-old was 36% [6]. However, mortality varies between different countries, with reports ranging from 8 to 36% [2,3,4,5,6]. This is probably due to different health care systems, different population characteristics, extent of laboratory tests and even preparedness for the pandemic (northern Italy was afflicted severely during the beginning of the pandemic with numbers that saturated their health care system). The mortality of 20.8% in our cohort of patients older than 65 years old with COVID-19 is consistent with that found in other countries. In addition, within this age group, older patients are at an increased risk of mortality.

With increasing age, comorbidities become more prevalent, which leads to higher susceptibility of developing severe infection [31,32]. Comorbidities such as CVD, hypertension, diabetes, COPD and CKD have been associated with the development of severe disease [11,19,33,34,35,36].

Patients with comorbidities such as hypertension, CVD, diabetes and CKD are likely to be treated with RAAS inhibitors, which is thought to upregulate the ACE 2 receptor, according to animal models [37,38,39]. In order to enter cells and consequently to allow its infection, SARS-Cov-2 needs to bind to the angiotensin-converting enzyme 2 (ACE 2) receptor present in alveolar cells [40,41]. Concern was raised that patients taking these drugs were at an increased risk of being infected with COVID-19 and at an increased risk of mortality, due to the upregulation of the ACE 2 receptor, allowing for a greater entry of the virus [12]. However, there is a lack of studies showing effects of RAAS inhibitors on the expression of ACE 3 receptors in the lungs and doses used in animal studies were much higher [42]. It was also hypothesized that the use of RAAS inhibitors might have potential benefits in COVID-19. Following the binding of SARS-Cov-2 to the ACE 2 receptor, there is a downregulation of this receptor expression on the lungs. This results in RAAS activation, contributing to acute lung injury and eventually leading to acute respiratory distress syndrome [42,43]. Despite this concern, specialists and international guidelines suggested against discontinuation of these medications [44,45,46].

Trecarichi et al. found no difference in in-hospital mortality, in a cohort of 50 patients with a mean age of 80 ± 12 years old and a 32% mortality rate, between patients treated with RAAS inhibitors and those not treated with these drugs (HR 0.50; 95% CI 0.14–1.81) [5]. Similar results were reported in a meta-analysis by Zhang et al. where the use of RAAS inhibitors did not increase the risk of COVID-19 infection, disease severity or mortality [47]. After analyzing 12 articles with over 19000 patients, four of them with a mean age greater than 65 years, it even concluded that patients receiving such medications indicated for hypertension treatment had a lower risk of mortality [47]. In another meta-analysis, which included 7 reports with almost 17000 patients with mortality rates ranging from 8 to 25%, by Nunes, however, ACE inhibitors use was associated with increased mortality in patients with COVID-19 infection [48]. In only one of these cohorts mean age was above 65 years. Nevertheless, work from Bravi et al., after analyzing a cohort of 1603 patients with a mortality of 9.6%, stated that, when restricted to hypertensive patients, RAAS inhibitors use was not associated with very severe or lethal disease (OR 0.87; 95% CI 0.50–1.49) [49].

Results are conflicting and, given the impossibility of randomized clinical trials on such matter, data must be obtained through observational studies, where causality is difficult to prove. RAAS inhibitors seem to pose no threat in hypertensive patients, although results regarding its use for other indications are conflicting. To the best of our knowledge, our study is the first to report a protective effect of these drugs in this subset of patients. We compared patients on these drugs with patients not taking these medications and there were no significant differences, which supports the results obtained. Hypertension and diabetes were more common in those on RAAS inhibitors, as it was expected.

Mortality in this population remains high and clues on the clinical course would be important for clinicians to adopt preventive measures in such patients. Laboratory parameters may potentially screen such at-risk patients of adverse outcomes. Other than interleukin-6, an expensive analysis, D-dimer, hypernatremia and lymphopenia, few other parameters have been described as associated with adverse outcomes [50,51]. Serum ferritin has already been shown to be increased during the worsening of the disease, as associated with the excessive inflammation seen during the cytokine storm, providing an indication of the risk of mortality [20]. Cecconi et al. revealed it could aid the early identification of patients at risk of clinical deterioration [52]. Hyperferritinemia has also been associated with admission to ICU and mortality [53]. These results are further supported by those in our study. To the best of our knowledge, high lactate levels at admission have not been reported as associated with mortality in COVID-19. The results of our study support that higher serum ferritin and lactate levels at admission are independent predictors of mortality. Both laboratory parameters are widely accessible and could help clinicians in the early identification of patients at risk of adverse outcomes.

Our study has some limitations. It is a retrospective study, so causality cannot be proven and randomized clinical trials would be necessary to validate the protective effect found with RAAS inhibition. In addition, the reason why patients were on such medications was not taken into consideration and we know it might affect results. Some missing laboratory data precluded to include parameters, such as D-dimers, which are known to be risk factors for COVID-19 mortality, in the multivariate regression analysis. Another limitation is the lack of available data on other potentially confounding factors, such as ethnicity. We did not analyze the difference in mortality between angiotensin converting enzyme inhibitor and angiotensin receptor blocker.

Despite these limitations, our study has several noteworthy virtues. It is the first study to analyze the effect of RAAS inhibitors use and mortality in elderly adults with COVID-19. This study has a reasonable population size and the population studied represents an incident cohort, so analysis of outcome predictors is more reliable.

## 5. Conclusions

Elderly patients are more susceptible to infection and adverse outcomes in COVID-19. In addition, higher serum ferritin and lactate levels at admission were found to be independent predictors of mortality and might act as early predictors of worsening disease in clinical practice. The results of our study suggest that chronic use of RAAS inhibitors might be protective as almost half of our patients were on this medication and had better survival, supporting the statement by international guidelines in not discontinuing such drugs in this population.

## Figures and Tables

**Table 1:** Patients’ baseline characteristics and in-hospital mortality.

Characteristic	Total (n = 346)	Survival (n = 274)	Mortality (n = 72)	*p*-Value
Age (years)	80.9 ± 8.7	79.8 ± 8.4	84.8 ±8.8	0.000
Gender (male)—n (%)	164 (47.4)	127 (46.4)	37 (51.4)	0.446
Co-morbidities—n (%)				
Hypertension	279 (80.6)	219 (79.9)	60 (83.3)	0.515
Diabetes	113 (32.7)	89 (32.5)	24 (33.3)	0.891
CVD	161 (46.5)	119 (43.4)	42 (58.3)	0.024
Heart failure	127 (36.7)	92 (33.6)	35 (48.6)	0.019
COPD	51 (14.7)	41 (15.0)	10 (13.9)	0.819
Cirrhosis	10 (2.9)	8 (2.9)	2 (2.8)	0.945
Neoplasia	53 (15.3)	36 (13.1)	17 (23.6)	0.028
CKD	98 (28.3)	66 (24.1)	32 (44.4)	0.000
CKD G5D	15 (15.3)	9 (13.6)	6 (18.75)	0.156
RAAS inhibitors—n (%)	171 (49.4)	144 (52.6)	27 (37.5)	0.043
Baseline sCr (mg/dL)	1.0 ± 0.5	1.0 ± 0.4	1.2 ± 0.8	0.010
Baseline eGFR (mL/min/1.73 m^2^)	65.3 ± 21.4	67.0 ± 20.6	58.7 ± 23.2	0.004
Brescia score >2—n (%)	11 (3.2)	8 (2.9)	3 (4.2)	0.024
Laboratory				
Admission sCr (mg/dL)	1.5 ± 1.4	1.4 ± 1.4	1.8 ± 1.5	0.094
Hemoglobin (g/dL)	12.3 ± 2.3	12.5 ± 2.1	11.7 ± 2.5	0.009
Anemia—n (%)	172 (49.7)	129 (47.1)	43 (59.7)	0.060
N/L ratio	7.0 ± 6.5	6.4 ± 6.1	9.1 ± 7.6	0.001
Hypoalbuminemia—n (%)	190 (54.9)	149 (54.4)	41 (56.9)	0.557
Serum ferritin (µg/dL)	1113.1 ± 1731.4	850.0 ± 713.1	2068.4 ± 3331.8	0.000
CRP (mg/dL)	10.4 ± 10.0	10.2 ± 10.1	10.9 ± 9.6	0.584
Acidemia—n (%)	31 (9.0)	21 (7.7)	10 (13.9)	0.114
Lactate levels (mg/dL)	15.0 ± 10.4	13.9 ± 7.4	19.2 ± 17.0	0.000
LDH (mg/dL)	333.3 ± 171.5	319.8 ± 134.0	385.8 ± 267.2	0.004
Nephrotoxins—n (%)	44 (12.7)	37 (13.5)	7 (9.7)	0.412
ICU admission—n (%)	68 (19.7)	55 (20.1)	13 (18.1)	0.692
Mechanical ventilation—n (%)	40 (11.6)	32 (11.7)	8 (11.1)	0.751
RRT—n (%)	43 (12.4)	31 (11.3)	12 (16.7)	0.627
Vasopressor use—n (%)	13 (3.8)	11 (4.0)	2 (2.8)	0.570
COVID-19 treatment				
Hydroxychloroquine—n (%)	91 (26.3)	72 (26.3)	19 (26.4)	0.897
Lopinavir/ritonavir—n (%)	116 (33.5)	98 (35.8)	18 (25)	0.094
Tocilizumab—n (%)	4 (1.2)	4 (1.5)	0 (0)	0.309
Corticosteroids—n (%)	83 (24.0)	72 (26.3)	11 (15.3)	0.069
Remdesivir—n (%)	25 (7.2)	23 (8.4)	2 (2.8)	0.111
LOS in hospital (days)	34.3 ± 46.3	35.6 ± 46.3	29.2 ± 46.4	0.300
In-hospital mortality—n (%)	72 (20.8)			

**Table 2:** Patients’ baseline characteristics according to baseline medication.

Characteristic	On RAAS Inhibitors (n = 171)	Not on RAAS Inhibitors (n = 175)	*p*-Value
Age (year)	81.5 ± 7.6	80.2 ± 9.7	0.185
Gender (Male)—n (%)	74 (43.3)	87 (49.7)	0.159
Co-morbidities—n (%)			
Hypertension	157 (91.8)	118 (67.4)	0.000
Diabetes	66 (38.6)	47 (26.9)	0.029
CVD	73 (42.7)	86 (49.1)	0.159
Heart failure	68 (39.8)	58 (33.1)	0.262
COPD	32 (18.7)	17 (9.7)	0.021
Cirrhosis	7 (4.1)	3 (1.7)	0.202
Neoplasia	22 (12.9)	29 (16.6)	0.288
CKD	45 (26.3)	51 (29.1)	0.470
CKD G5D	2 (4.4)	13 (25.4)	0.009
Baseline SCr (mg/dL)	1.0 ± 0.5	1.1 ± 0.5	0.380
Baseline eGFR (mL/min/1.73 m^2^)	65.5 ± 21.5	65.3 ± 21.4	0.927
Brescia Score >2—n (%)	33 (19.3)	25 (14.3)	0.534
Laboratory			
Admission SCr (mg/dL)	1.5 ± 1.7	1.5 ± 1.4	0.895
Hemoglobin (g/dL)	12.4 ± 2.2	12.2 ± 2.4	0.587
Anemia—n (%)	80 (46.8)	90 (51.4)	0.303
N/L ratio	7.5 ± 7.6	6.5 ± 5.3	0.145
Hypoalbuminemia—n (%)	95 (55.6)	94 (53.7)	0.771
Serum ferritin (µg/dL)	970.9 ± 1139.4	1275.1 ± 2211.6	0.253
CRP (mg/dL)	10.3 ± 10.9	10.6 ± 9.0	0.745
Acidemia—n (%)	16 (9.4)	15 (8.6)	0.807
Lactate levels (mg/dL)	16.2 ± 12.5	13.9 ± 7.7	0.059
LDH (mg/dL)	336.6 ± 199.3	331.4 ± 139.8	0.782
Nephrotoxins—n (%)	20 (11.7)	23 (13.1)	0.625
ICU admission—n (%)	37 (21.6)	30 (17.1)	0.354
Mechanical ventilation—n (%)	21 (12.3)	18 (10.3)	0.638
Vasopressor use—n (%)	5 (2.9)	7 (4.0)	0.540
LOS in hospital (days)	28.7 ± 33.1	21.0 ± 17.1	0.109
In-hospital mortality—n (%)	27 (15.8)	42 (24.0)	0.043

**Table 3:** Univariate and multivariate analysis of factors predictive of mortality in COVID-19 patients.

Characteristic	Mortality			
	Unadjusted OR (95% CI)	*p* Value	Adjusted OR (95% CI)	*p* Value
Age	1.08 (1.04–1.11)	0.000	1.11 (1.04–1.18)	0.002
Gender (male)	1.22 (0.73–2.06)	0.446		
Co-morbidities				
Hypertension	1.27 (0.63–2.50)	0.516		
Diabetes	1.04 (0.60–1.80)	0.891		
CVD	1.82 (1.08–3.09)	0.025	1.01 (0.36–2.78)	0.993
Heart failure	1.87 (1.11–3.17)	0.019	1.88 (0.70–5.03)	0.208
CKD	2.73 (1.58–4.72)	0.000	1.07 (0.38–3.03)	0.900
COPD	0.92 (0.44–1.93)	0.819		
Cirrhosis	0.95 (0.20–4.56)	0.945		
Neoplasia	2.04 (1.07–3.90)	0.030	2.12 (0.50–8.94)	0.309
RAAS inhibitors	0.58 (0.34–0.99)	0.045	0.27 (0.10–0.75)	0.011
Admission sCr	1.13 (0.97–1.30)	0.113		
Hemoglobin	0.86 (0.76–0.96)	0.010	0.83 (0.66–1.06)	0.135
N/L ratio	1.06 (1.02–1.09)	0.003	0.97 (0.88–1.06)	0.455
Hypoalbuminemia	1.24 (0.61–2.53)	0.558		
Serum ferritin	1.00 (1.00–1.00)	0.003	1.00 (1.00–1.00)	0.003
CRP	1.01 (0.98–1.03)	0.583		
Acidemia	1.90 (0.85–4.24)	0.119		
LDH	1.00 (1.00–1.00)	0.014	1.00 (1.00–1.01)	0.415
Lactate levels	1.05 (1.02–1.08)	0.001	1.08 (1.02–1.14)	0.006
Nephrotoxins	0.70 (0.30–1.65)	0.414		
Brescia score	1.26 (0.98–1.62)	0.068		
AKI	2.33 (1.22–4.47)	0.011	1.92 (0.59–6.33)	0.282
ICU admission	0.87 (0.45–1.71)	0.692		
Mechanical ventilation	0.88 (0.38–2.00)	0.751		
RRT	1.12 (0.53–2.36)	0.769		

## Data Availability

The datasets generated during and/or analyzed during the current study are available from the corresponding author on reasonable request.

## References

[1] WHO (2021). COVID-19 Weekly Epidemiological Update.

[2] Shahid Z., Bs R.K., Bs B.M., Kepko D., Bs D.R., Patel R., Mbbs C.S.A., Vunnam R.R., Sahu N., Bhatt D. (2020). COVID-19 and Older Adults: What We Know. J. Am. Geriatr. Soc..

[3] Wu Z., McGoogan J.M. (2020). Characteristics of and Important Lessons from the Coronavirus Disease 2019 (COVID-19) Outbreak in China: Summary of a Report of 72 314 Cases from the Chinese Center for Disease Control and Prevention. JAMA.

[4] Heras E., Garibaldi P., Boix M., Valero O., Castillo J., Curbelo Y., Gonzalez E., Mendoza O., Anglada M., Miralles J.C. (2021). COVID-19 mortality risk factors in older people in a long-term care center. Eur. Geriatr. Med..

[5] Trecarichi E.M., Mazzitelli M., Serapide F., Pelle M.C., Tassone B., Arrighi E., Perri G., Fusco P., Scaglione V., Davoli C. (2020). Clinical characteristics and predictors of mortality associated with COVID-19 in elderly patients from a long-term care facility. Sci. Rep..

[6] Grasselli G., Zangrillo A., Zanella A., Antonelli M., Cabrini L., Castelli A., Cereda D., Coluccello A., Foti G., Fumagalli R. (2020). Baseline Characteristics and Outcomes of 1591 Patients Infected with SARS-CoV-2 Admitted to ICUs of the Lombardy Region, Italy. JAMA.

[7] Wang Y., Lu X., Li Y., Chen H., Chen T., Su N., Huang F., Zhou J., Zhang B., Yan F. (2020). Clinical Course and Outcomes of 344 Intensive Care Patients with COVID. Am. J. Respir. Crit. Care Med..

[8] Chen R., Liang W., Jiang M., Guan W., Zhan C., Wang T., Tang C., Sang L., Liu J., Ni Z. (2020). Risk Factors of Fatal Outcome in Hospitalized Subjects With Coronavirus Disease 2019 From a Nationwide Analysis in China. Chest.

[9] Du R.-H., Liang L.-R., Yang C.-Q., Wang W., Cao T.-Z., Li M., Guo G.-Y., Du J., Zheng C.-L., Zhu Q. (2020). Predictors of mortality for patients with COVID-19 pneumonia caused by SARS-CoV-2: A prospective cohort study. Eur. Respir. J..

[10] Leung C. (2020). Risk factors for predicting mortality in elderly patients with COVID-19: A review of clinical data in China. Mech. Ageing Dev..

[11] McMichael T.M., Currie D.W., Clark S., Pogosjans S., Kay M., Schwartz N.G., Lewis J., Baer A., Kawakami V., Lukoff M.D. (2020). Epidemiology of Covid-19 in a Long-Term Care Facility in King County, Washington. N. Engl. J. Med..

[12] Gracia-Ramos A.E. (2020). Is the ACE2 Overexpression a Risk Factor for COVID-19 Infection?. Arch. Med Res..

[13] Steinberg E., Balakrishna A., Habboushe J., Shawl A., Lee J. (2020). Calculated decisions: COVID-19 calculators during extreme resource-limited situations. Emerg. Med. Pract..

[14] Khwaja A. (2012). KDIGO Clinical Practice Guidelines for Acute Kidney Injury. Nephron.

[15] (2008). American Diabetes Association Standards of Medical Care in Diabetes. Diabetes Care.

[16] Council E.S., Redon J., Narkiewicz K., Nilsson P.M., Burnier M., Viigimaa M., Ambrosioni E., Coca A., Olsen M.H., Schmieder R.E. (2013). 2013 ESH/ESC Guidelines for the management of arterial hypertension. Eur. Heart J..

[17] Gonzalez A.L., Waddell L.S. (2016). Blood Gas Analyzers. Top. Companion Anim. Med..

[18] Wang L., He W., Yu X., Hu D., Bao M., Liu H., Zhou J., Jiang H. (2020). Coronavirus disease 2019 in elderly patients: Characteristics and prognostic factors based on 4-week follow-up. J. Infect..

[19] Yanez N.D., Weiss N.S., Romand J.-A., Treggiari M.M. (2020). COVID-19 mortality risk for older men and women. BMC Public Health.

[20] Zhou F., Yu T., Du R., Fan G., Liu Y., Liu Z., Xiang J., Wang Y., Song B., Gu X. (2020). Clinical course and risk factors for mortality of adult inpatients with COVID-19 in Wuhan, China: A retrospective cohort study. Lancet.

[21] Porcheddu R., Serra C., Kelvin D., Kelvin N., Rubino S. (2020). Similarity in Case Fatality Rates (CFR) of COVID-19/SARS-COV-2 in Italy and China. J. Infect. Dev. Ctries..

[22] Svartengren M., Philipson K., Falk R. (2005). Long-term clearance from small airways decreases with age. Eur. Respir. J..

[23] Levitzky M.G. (1984). Effects of aging on the respiratory system. Physiologist.

[24] Ho J., Chan K.N., Hu W.H., Lam W.K., Zheng L., Tipoe G.L., Sun J., Leung R., Tsang K.W. (2001). The Effect of Aging on Nasal Mucociliary Clearance, Beat Frequency, and Ultrastructure of Respiratory Cilia. Am. J. Respir. Crit. Care Med..

[25] Kang S.-J., Jung S.I. (2020). Age-Related Morbidity and Mortality among Patients with COVID-19. Infect. Chemother..

[26] Perrotta F., Corbi G., Mazzeo G., Boccia M., Aronne L., D’Agnano V., Komici K., Mazzarella G., Parrella R., Bianco A. (2020). COVID-19 and the elderly: Insights into pathogenesis and clinical decision-making. Aging Clin. Exp. Res..

[27] Castle S.C. (2000). Clinical Relevance of Age-Related Immune Dysfunction. Clin. Infect. Dis..

[28] Tay M.Z., Poh C.M., Rénia L., Macary P.A., Ng L.F.P. (2020). The trinity of COVID-19: Immunity, inflammation and intervention. Nat. Rev. Immunol..

[29] Bonafè M., Prattichizzo F., Giuliani A., Storci G., Sabbatinelli J., Olivieri F. (2020). Inflamm-aging: Why older men are the most susceptible to SARS-CoV-2 complicated outcomes. Cytokine Growth Factor Rev..

[30] Aw D., Silva A.B., Palmer D.B. (2007). Immunosenescence: Emerging challenges for an ageing population. Immunology.

[31] Fryar C.D., Ostchega Y., Hales C., Zhang G., Kruszon-Moran D. (2017). Hypertension Prevalence and Control among Adults: United States, 2015–2016. NCHS Data Brief.

[32] Centers for Disease Control and Prevention (2020). National Diabetes Statistics Report, 2020.

[33] Huang C., Wang Y., Li X., Ren L., Zhao J., Hu Y., Zhang L., Fan G., Xu J., Gu X. (2020). Clinical features of patients infected with 2019 novel coronavirus in Wuhan, China. Lancet.

[34] Jin J.-M., Bai P., He W., Wu F., Liu X.-F., Han D.-M., Liu S., Yang J.-K. (2020). Gender Differences in Patients With COVID-19: Focus on Severity and Mortality. Front. Public Health.

[35] Li X., Xu S., Yu M., Wang K., Tao Y., Zhou Y., Shi J., Zhou M., Wu B., Yang Z. (2020). Risk factors for severity and mortality in adult COVID-19 inpatients in Wuhan. J. Allergy Clin. Immunol..

[36] Imam Z., Odish F., Gill I., O’Connor D., Armstrong J., Vanood A., Ibironke O., Hanna A., Ranski A., Halalau A. (2020). Older age and comorbidity are independent mortality predictors in a large cohort of 1305 COVID-19 patients in Michigan, United States. J. Intern. Med..

[37] Zheng Y.-Y., Ma Y.-T., Zhang J.-Y., Xie X. (2020). COVID-19 and the cardiovascular system. Nat. Rev. Cardiol..

[38] Igase M., Kohara K., Nagai T., Miki T., Ferrario C.M. (2008). Increased Expression of Angiotensin Converting Enzyme 2 in Conjunction with Reduction of Neointima by Angiotensin II Type 1 Receptor Blockade. Hypertens. Res..

[39] Ferrario C.M., Jessup J., Chappell M.C., Averill D.B., Brosnihan K.B., Tallant E.A., Diz D.I., Gallagher P.E. (2005). Effect of Angiotensin-Converting Enzyme Inhibition and Angiotensin II Receptor Blockers on Cardiac Angiotensin-Converting Enzyme. Circulation.

[40] Zhou P., Yang X.-L., Wang X.-G., Hu B., Zhang L., Zhang W., Si H.-R., Zhu Y., Li B., Huang C.-L. (2020). A pneumonia outbreak associated with a new coronavirus of probable bat origin. Nature.

[41] Wu F., Zhao S., Yu B., Chen Y.-M., Wang W., Song Z.-G., Hu Y., Tao Z.-W., Tian J.-H., Pei Y.-Y. (2020). A new coronavirus associated with human respiratory disease in China. Nature.

[42] Albashir A.A.D. (2021). Renin-Angiotensin-Aldosterone System (RAAS) Inhibitors and Coronavirus Disease 2019 (COVID-19). South. Med. J..

[43] Kuba K., Imai Y., Rao S., Gao H., Guo F., Guan B., Huan Y., Yang P., Zhang Y., Deng W. (2005). A crucial role of angiotensin converting enzyme 2 (ACE2) in SARS coronavirus–induced lung injury. Nat. Med..

[44] Trifirò G., Pharmacology T.I.S.O., Crisafulli S., Andò G., Racagni G., Drago F. (2020). Should Patients Receiving ACE Inhibitors or Angiotensin Receptor Blockers be Switched to Other Antihypertensive Drugs to Prevent or Improve Prognosis of Novel Coronavirus Disease 2019 (COVID-19)?. Drug Saf..

[45] De Simone G. (2020). Position Statement of the ESC Council on Hypertension on ACE-Inhibitors and Angiotensin Receptor Blockers. Eur. Soc. Cardiol..

[46] Rico-Mesa J.S., White A., Anderson A. (2020). Outcomes in Patients with COVID-19 Infection Taking ACEI/ARB. Curr. Cardiol. Rep..

[47] Zhang X., Yu J., Pan L.Y., Jiang H.Y. (2020). ACEI/ARB use and risk of infection or severity or mortality of COVID-19: A systematic review and meta-analysis. Pharmacol. Res..

[48] Nunes J.P.L. (2020). Mortality and use of angiotensin-converting enzyme inhibitors in COVID 19 disease: A systematic review. Porto Biomed. J..

[49] Bravi F., Flacco M.E., Carradori T., Volta C.A., Cosenza G., De Togni A., Martellucci C.A., Parruti G., Mantovani L., Manzoli L. (2020). Predictors of severe or lethal COVID-19, including Angiotensin Converting Enzyme inhibitors and Angiotensin II Receptor Blockers, in a sample of infected Italian citizens. PLoS ONE.

[50] Gao Y., Li T., Han M., Li X., Wu D., Xu Y., Zhu Y., Liu Y., Wang X., Wang L. (2020). Diagnostic utility of clinical laboratory data determinations for patients with the severe COVID-19. J. Med. Virol..

[51] Tan L., Wang Q., Zhang D., Ding J., Huang Q., Tang Y.Q., Wang Q., Miao H. (2020). Correction: Lymphopenia predicts disease severity of COVID-19: A descriptive and predictive study. Signal. Transduct. Target. Ther..

[52] Cecconi M., Piovani D., Brunetta E., Aghemo A., Greco M., Ciccarelli M., Angelini C., Voza A., Omodei P., Vespa E. (2020). Early Predictors of Clinical Deterioration in a Cohort of 239 Patients Hospitalized for Covid-19 Infection in Lombardy, Italy. J. Clin. Med..

[53] Cheng L., Li H., Li L., Liu C., Yan S., Chen H., Li Y. (2020). Ferritin in the coronavirus disease 2019 (COVID-19): A systematic review and meta-analysis. J. Clin. Lab. Anal..

